# Medical screening in dental settings: a qualitative study of the views of authorities and organizations

**DOI:** 10.1186/s13104-015-1543-8

**Published:** 2015-10-19

**Authors:** Göran Friman, Margareta Hultin, Gunnar H. Nilsson, Inger Wårdh

**Affiliations:** Department of Dental Medicine and Academic Centre of Gerodontics, Karolinska Institutet, Stockholm, Sweden; Department of Health and Environmental Sciences, Karlstad University, Karlstad, Sweden; Department of Dental Medicine/Division of Periodontology, Karolinska Institutet, Huddinge, Sweden; Department of Neurobiology, Care Sciences and Society, Karolinska Institutet, Huddinge, Sweden

**Keywords:** Diabetes, Hypertension, Medical screening, Position of authorities and organizations

## Abstract

**Background:**

The practice of identifying individuals with undiagnosed diabetes mellitus type II or undiagnosed hypertension by medical screening in dental settings has been received positively by both patients and dentistry professionals. This identification has also shown to be cost-effective by achieving savings and health benefits, but no investigation has been made of the attitudes of authorities and organizations. The aim of this study was to describe the views of authorities and organizations.

**Results:**

Thirteen authorities and organizations were interviewed of the sample of 20 requested. Seven approached authorities and organizations did not believe it was relevant to participate in the study. The manifest analysis resulted in four categories: medical screening ought to be established in the society; dentistry must have relevant competence to perform medical screening; medical screening requires cooperation between dentistry and health care; and dentistry is not the only context where medical screening could be performed. The latent analysis resulted in an emerging theme: positive to, but uncertain about, the concept of medical screening in dental settings. The spokespersons for the approached authorities and organizations had a positive view of medical screening but the respondents experienced a lack of facts concerning the scientific communities’ position, guidelines and procedures in the topic.

**Conclusions and implications:**

Approached authorities and organizations generally had a positive view of medical screening in dental settings but were uncertain about the concept. Further scientific knowledge and guidelines concerning the topic are needed before it can be commonly introduced and additional research on implementation strategies and long-term follow-up of medical screening are needed.

## Background

Combined with increasing longevity, the population has become more frail and thereby more vulnerable to both oral and general diseases [1]. Diabetes mellitus type II [2] and cardiovascular diseases (CVD) such as hypertension [3] are among the most common global health scourges.

These diseases are usually not detected until complications arise, for example vascular damages, angina or myocardial infarction [4, 5]. In a review article from Cochrane [6], the authors suggest that future research should focus on, for example, screening for cardiovascular risk factors, chronic obstructive pulmonary disease, diabetes, or kidney disease. Future research is needed to find the most optimal way to screen for these common diseases to optimize the benefit and cost-effectiveness [7].

The interest in medical screening in dental settings during the early 2000s has been extensive [8–11], and the activities to identify individuals with undiagnosed diabetes mellitus type II or undiagnosed hypertension have been positively received by patients and dentistry, and have shown to be cost-effective by achieving savings and health benefits.

Several studies were conducted to evaluate medical screening in dental settings [8–11] as associations have been pointed out between periodontal and cardiovascular diseases as well as diabetes [12–14]. These results indicate that medical screening in dental settings could be an effective component of disease prevention and enhance cross-border cooperation between dental and medical care [15].

Dentistry has a tradition of working in a prophylactic manner to prevent diseases through customized regular examinations and oral health care programs [16, 17]. This is one of the reasons why medical screening would be suitable for dental care.

The benefits of medical screening in dental settings for early diagnosis and identification of patients at risk are documented [18]. In the face of a more general implementation, dentists’ attitudes have been examined. Dentists considered that medical screening could be vital in a public health perspective and were willing to incorporate it into their routines, but additional education and training were necessary before an implementation [18].

Studies of the patients’ attitudes toward screening for medical conditions in a dental setting have also been conducted and most patients expressed a favorable attitude toward chairside screening [19]. They also expressed the importance of the dental professionals having the necessary medical knowledge and, when appropriate, referring to the health care service [20]. No investigation, however, had yet been conducted on the attitudes of authorities and organizations, which could have opinions about medical screening in dental settings concerning patient perspective, professional competence, and quality of care and education, or which might have a potential role in the eventual implementation of medical screening. Thus the aim of this study was to describe the view of authorities and organizations in a Swedish context.

## Methods

Both quantitative and qualitative data were collected through a standardized questionnaire and subsequent interviews, but the study was mainly based on qualitative data [21].

The study was approved by the Regional Ethical Review Board in Uppsala, Sweden, in accordance with the ethical standards and with the Helsinki Declaration of 1975, as revised in 1983. The participants received both verbal and written information about the study, they were not compensated for their time, and their participation was completely voluntary.

### Study setting and population

Through a purposive sampling approach [22], 20 Swedish authorities and organizations were contacted. The research team initially sought contact with the chairman, vice chairman or spokesperson. If none of these chief persons were able respond or were unavailable for an interview, another representative was requested as respondent. The research team considered the selection to be relevant to meet the purpose of the study. Thirteen participating authorities and organizations took part in the study and are described in Table 1.

**Table 1:** Swedish authorities and organizations that took part in the study

Authorities	Organizations
The Dental Board of the County Council of Värmland (*Tandvårdsnämnden, Landstinget I Värmland*)	The Swedish Medical Association (*Sveriges Läkarförbund*)
	The Swedish Association of Dental Hygienists (*Sveriges Tandhygienistförening*)
	Swedish Dental Nursing Association (*Svenska Tandsköterskeförbundet*)
	The Swedish Association of Health Professionals (*Vårdförbundet*)
	The Uppsala Dental Service Organisation (*Folktandvårdsföreningen, Folktandvårdens kansli Uppsala*)
	Praktikertjänst AB
	SKL—Swedish Association of Local Authorities and Regions (*SKL*—*Sveriges kommuner och landsting*)
	Swedish Diabetes Association (*Svenska diabetesförbundet*)
	The Swedish Stroke Association (*STROKE*-*Riksförbunde*t)
	Faculty of Odontology at Malmö University
	Institute of Odontology, The Sahlgrenska Academy, Gothenburg
	Department of Dental Medicine, Karolinska Institute, Huddinge

The Swedish names of authorities and organizations given in parentheses

The selected authorities and organizations were contacted primarily by phone and those who gave consent to take part in the study got an email with further information and a questionnaire.

### Data collection

Of the twenty purposively sampled Swedish authorities and organizations, there were seven dropouts. The reasons given for not participating were: not taken a position on the issue, not relevant instance, and not suitable as they work exclusively with issues regarding primary and secondary education. One respondent did not reply. Thirteen Swedish authorities and organizations constituted the final study material.

All respondents received a standardized questionnaire with eighteen questions concerning medical screening in dental settings to gain insight into the topic and prepare for an interview, as they were mainly supposed to express positive and negative opinions that characterized their authority or organization and not personal thoughts. The questionnaire design was first evaluated in a pilot study involving ten selected teachers at the Department of Dental Medicine, Karolinska Institutet, Huddinge. Using a traditional four-point Likert scale, respondents indicated to what degree a statement was consistent with their opinion by stating “Strongly disagree”, “Disagree”, “Agree” or “Strongly agree” [23].

Qualitative data were collected through interviews by two trained calibrated interviewers with thirteen open-ended questions from an interview guide that addressed the relevant topics (Table 2). The guide was tested on a pilot person to optimize the questions and to get as comprehensive answers as possible. Each open-ended question had follow-up questions so as to, if possible, achieve saturation. The main portion of the interviews was conducted by phone and the rest during personal appointments at offices of the authorities and organizations from December 2012 to April 2013.

**Table 2:** Interview guide

Issues
Territorial mentality
Voluntariness of the patients, the dental care and the health care
Patient’s perspective, integrity, confidentiality, availability, quality of life
Quality of care and caring responsibilities
Competence, the lowest level of care for risk assessment
Education and qualifications
Economics
Legal affairs

Each interview lasted approximately 40 min and was audio-recorded and transcribed verbatim. After 13 interviews, the authors concluded that no new relevant information emerged, saturation was reached and the data collection ended [24].

### Data analysis

The qualitative data were analyzed with qualitative manifest and latent content analysis [24]. The interview texts were read in their entirety, discussed by the authors and divided into groups of meaning-bearing units, codes. Similar codes were merged and then sorted into subcategories and categories. A comparison was made with the interview guide to see if the categories corresponded to the question areas. This represented the manifest level of analysis. The authors then looked for a main category or an underlying theme, the latent content analysis. The qualitative results were illustrated with quotations. The quotes presented were expressed by the interviewees and were used to exemplify the categories and theme.

## Results

### The quantitative data

The quantitative data was analyzed descriptively. The answers on the 18 items resulted in 46 % (108) positive responses to medical screening in dental settings, 41 % (95) negative responses and 13 % (31) non-responses.

### The qualitative data

**Fig. 1:**
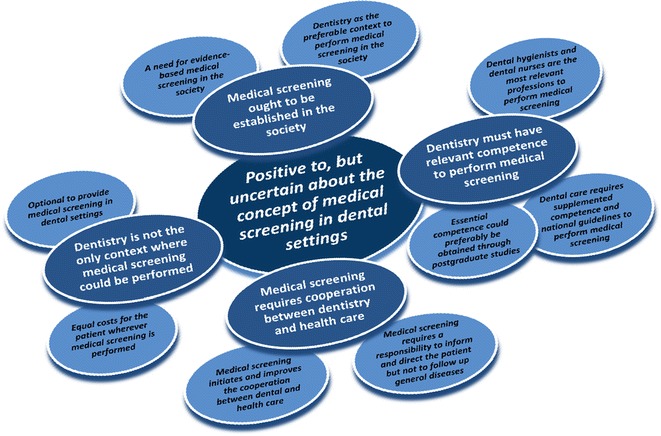
Graphical presentation of the descriptive content analysis, subcategories, categories and theme

Meaning-bearing units were extracted from the texts, and subcategories and categories were formed (Table 3). Figure 1 gives a visual presentation of the findings. The analysis revealed four categories made up of nine subcategories. One theme permeated them all. Below, these results are presented in greater detail with citations.

**Table 3:** Summary of subcategories and categories

Subcategory	Category
Dentistry as the preferable context to perform medical screening in the society A need for evidence-based medical screening in the society	Medical screening ought to be established in the society
Dental hygienists and dental nurses are the most relevant professions to perform medical screening Dental care requires supplemented competence and national guidelines to perform medical screening Essential competence could preferably be obtained through postgraduate studies	Dentistry must have relevant competence to perform medical screening
Medical screening requires a responsibility to inform and direct the patient but not to follow up general diseases Medical screening initiates and improves the cooperation between dental and health care	Medical screening requires cooperation between dentistry and health care
Equal costs for the patient wherever medical screening is performed Optional to provide medical screening in dental settings	Dentistry is not the only context where medical screening could be performed

### Manifest descriptive content analysis

#### Medical screening ought to be established in the society

##### Dentistry as the preferable context to perform medical screening in the society

The majority of the participants pointed out the importance of medical screening as a community-promoting action. They argued that dental care should take that responsibility, due to the fact that dental care follows its patients more frequently on a regular basis in comparison with health care.

Because we can see basically the entire healthy population fairly regularly in dental care, it can be an advantage to identify a number of illnesses that could take a while to diagnose. For these, it could be a great advantage if this were the case.

Those who were more reluctant stressed the difficulties of making an overall assessment and identifying at-risk individuals. A lack of resources in dentistry, skills shortages and sources of error in procedural techniques were mentioned as stumbling blocks. Territorial thinking was also highlighted as a possible problem. The predominant portion of participants, however, agreed that these problems could be overcome through increased competence.

… it’s still health care that owns this issue. So it has to be done in very close collaboration with health care, so that there is a, *um*, big picture for the patient.

##### A need for evidence-based medical screening in the society

A minor portion of the participants answered that medical screening was needed in our society, but stressed the need for common guidelines concerning competence, skills and patient management. The lack of evidence was also the main reason for not looking upon medical screening as something valuable. A few participants considered the need not equivalent to the cost.

#### Dentistry must have relevant competence to perform medical screening

##### Dental hygienists and dental nurses are the most relevant professions to perform medical screening

Most participants made the judgment that dental care has the personnel to implement medical screening. Dental hygienists or dental nurses were considered to be the most suitable personnel group for the performance of medical screening in dental settings. Medical screening can be a natural part of the hygienist’s health conversation with their patients. Some participants argued that there is already a shortage of capacity in dentistry and the possible introduction of medical screening means increased need for resources.

I think that we can develop the skills of a category of personnel in dentistry to perform this. A dental hygienist can be responsible for this after further training.

##### Dental care requires supplemented competence and national guidelines to perform medical screening

The majority of the participants answered that there was insufficient expertise in dental care to perform medical screening and that competence needed to be supplemented. Other participants clarified their answer by saying that dental care was capable of performing the investigations but lacked the skills to interpret the test results. One association brought up the opinion that dental care did not need specific regulations to govern the conduct of medical screening. There are already regulations and national guidelines in place to regulate various types of screening, but not specific regulations and guidelines for medical screening in dental settings, which would be demanded.

Yes, I think that you can probably always in these situations have some form of regulations and guidelines. I think that you should have these, because otherwise you can’t perform medical screening if it’s not regulated in some way.

##### Essential competence could preferably be obtained through postgraduate studies

Most participants agreed that basic education for both dentists and dental hygienists already contained a lot and that there was no space for new topics; however the competence could be obtained through postgraduate studies. One of the participants felt that the practical part should be during dental hygienists’ undergraduate studies. Others advocated a combination of undergraduate and postgraduate studies.

Competence could be increased in undergraduate studies, because all higher education should be adapted to potential future conditions. This knowledge could also be acquired in continuing education.

#### Medical screening requires cooperation between dentistry and health care

##### Medical screening requires a responsibility to inform and direct the patient but not to follow up general diseases

The participants agreed that dental care had the mission to identify patients at risk and the responsibility to inform the patient about the screening results, whether they were at risk of a medical disease or not, while emphasizing that dental care is not able to make medical diagnoses of general diseases. Dental care has an obligation to follow up the patient to a certain extent. That is, possibly referring the patient or recommending her to seek health care, and the follow-up should rest with health care. A necessity for effective cooperation is professional communication, through referrals.

Yes, following up on the patient is a must. You have to refer the patient for further treatment. This requires good collaboration with primary care.

… this assumes that there is a good communication between dentistry and health care so that the patient can be referred or transferred over to health care… The responsibility for continued care must lie with health care!

##### Medical screening initiates and improves the cooperation between dental and health care

The overwhelming majority of the participants answered that cooperation between dental and health care would improve health-promotion measures not only for the patients but also for society. The cooperation provides opportunities for evaluation of the at-risk groups who need to be screened, cut-off levels and referral management, and clear guidelines for implementation and communication. Misjudgments of sample values may result in increased workload for health care, a risk that can be reduced or possibly eliminated by collaboration.

Yes, this would certainly mean a greater workload when patients are referred who are not sick.

Yes, I would really like it if we could cooperate … there are only benefits to be gained by all of this, that’s for sure …

#### Dentistry is not the only context where medical screening could be performed

##### Equal costs for the patient wherever medical screening is performed

Concerning costs, the participants responded very evenly. The participants said that it was a difficult question to talk about because the benefit of medical screening was not scientifically documented yet, as far as they were aware. But it was judged to be fair to the patient to pay the same amount regardless of whether dental or health care performed a screening.

No, I think that the prerequisite for implementation of this is that there are no increased costs for the patient.

##### Optional to provide medical screening in dental settings

To the question of whether all dental settings should provide medical screening, just over half of the participants considered that there must be a care package that is optional and set by each individual dental clinic depending on the clinic’s interest. The remaining participants considered that all dental clinics should introduce medical screening. Introduction must be widespread to have a clear public health effect. The impact would not be as effective if medical screening did not reach out to the entire population.

In a dream world, I would say of course, but I know that they won’t do it because not everyone is interested in this.

### Latent content analysis: theme

#### Positive to, but uncertain about the concept of medical screening in dental settings

Approached authorities and organizations generally had a positive view of medical screening in dental settings but the responding spokespersons were uncertain about the concept, because they experienced a lack of facts concerning the scientific knowledge position, guidelines and procedures in the topic (Fig. 1).

… it must be based on what existing scientific evidence there is for medical screening actually reducing disease in the population and contributing to overall health. In other words, it must be based on existing medical screening actually having a positive effect, and if it does, well then it is obvious there should be clear rules governing it … Just like all women of a certain age are offered mammograms!

## Discussion

This study showed that the spokespersons were generally positive to medical screening in dental settings but requested more knowledge as they were uncertain about the concept.

### Method discussion

The quantitative questionnaire data were analyzed descriptively by means of respondents’ positive and negative attitudes towards medical screening. The aim was to make the respondents take a position on medical screening. A limitation appeared at this stage, as several respondents avoided answering. But this weakness was turned into a strength in the subsequent collection of qualitative data during the interviews, where respondents were asked why they previously avoided taking a stand, which resulted in a more comprehensive answer. However, this study should not be considered a report from Swedish authorities and organizations concerning medical screening, as some presumptive respondents did not wish to participate.

Achieving credibility is always important in all research, especially qualitative methods [24]. To ensure credibility [24], the interviewer was a person unknown to the respondents and who had limited pre-understanding of the subject. The respondents decided themselves where the interview should be conducted. Concerning the telephone interview alternative, there is a lack of additional information due to nonverbal reactions, but this was in some cases the only possibility of holding an interview, and a comparison of face-to-face and telephone interviews has revealed no significant differences in yielded results [25].

A limitation of the study was that the participants responded as representatives of Swedish authorities and organizations but partly with personal opinions. As a spokesperson for an authority or organization, it can be presumed that the spokesperson provides substantially representative answers, as official policy documents are not available. Neither should this study be taken for a total representative report from Sweden.

The transcriptions and analyses were made as quickly as possible after the qualitative data collection to optimize the interpretations and to secure relevant data in order to increase the dependability [24]. Accurate written descriptions of the research process and the context were prepared in order to pursue transferability [24] and to give the reader the ability to evaluate whether these findings are transferable to other similar Swedish or foreign contexts. When analyzing the collected data, a triangulation could be done to increase the reliability of the results through parallel analysis of the collected quantitative and qualitative data [26].

### Results discussion

Medical screenings in dental settings are showing an increasing trend, and in a study involving 28 dental practices in the US and Sweden, the Dental Practice-Based Research Network found that a significant proportion of tested dental patients had abnormal blood glucose values [27]. These results confirm the clinical relevance and specific challenge of investigating and describing the view of authorities and organizations in a Swedish context. To our knowledge no studies have been performed concerning this topic.

General health checks are evaluated in the Cochrane review article by Krogsbøll et al. [6], who aimed to quantify the benefits and harms of general health checks. The authors summarized with the words “general health checks in adults did not reduce morbidity or mortality”. This review article has been discussed in scientific literature and one weakness of the report is that most studies began in the 1960s and 1970s. Diagnosis and treatment methods may have changed over time and the report excluded studies involving only elderly individuals over 65 years of age [28]. All the reviewed studies also evaluated asymptomatic populations that were excluded for disease or risk factors. The authors suggested finally that future research should focus on, for example, screening for cardiovascular risk factors, chronic obstructive pulmonary disease, diabetes, or kidney disease, which opens the door for major studies [6].

By the early 2000s, it was already the trend in the US to make dental hygienists an integral part of the health care workforce [29]. As prevention specialists, part of their job was considered to be detecting the presence of general diseases. But there were educational requirements for medical screening and they did not make medical diagnoses.

The Swedish National Board of Health and Welfare in Sweden has emphasized the importance of primary preventive measures like medical screening (National Guidelines for Diabetes Care—summary, 2013, http://www.socialstyrelsen.se/nationalguidelines). “Health and medical care should carry out a screening of individuals who run an increased risk of developing diabetes type II, primarily in order to offer lifestyle changes.” These guidelines were not directed at dental care at this stage.

The aim of medical screening performed by the dental care service is early identification of patients at increased risk of developing coronary heart disease and diabetes mellitus, yet unaware of their increased risk [30]. The same authors also presented that dental settings could be a health promotion entry point into medical care for individuals not previously engaged with a primary care provider [30]. The cooperation between dental and medical care has proven to be an effective way to discover unknown hypertension [11, 31].

In their 2015 National Guidelines for Diabetes Care, the Swedish National Board of Health and Welfare recommended that health care refer to dental care individuals with diabetes with increased risk of impaired oral health or ongoing inflammatory disease of the tissues surrounding the teeth and dental implants. This cross-border communication is intended to result in decisions on treatment and preventive measures against caries, periodontitis and peri-implantitis. This guideline promotes an increased cooperation between dentistry and health care [32].

During the interviews it emerged that medical screening could be performed in many contexts, but experience indicates that routine opportunistic screening is easier to implement and more reliable than other screening programs that cannot leverage the existing infrastructures and human resources of formal medical settings [33]. Dental settings are therefore a natural choice of context alongside primary medical care.

Dentistry today is increasing cooperation with medical care regarding the treatment of other diseases that might be associated with hypertension, such as obstructive sleep apnea syndrome, OSAS [34, 35]. This further points to dentistry as a preferable context for medical screening.

Some of the respondents felt that an early diagnosis is always economically beneficial for society, but they had little knowledge of what savings could be achieved. The cost effectiveness of screening is important, and this effectiveness was studied in a Swedish population and found to depend on whether blood pressure screening was combined with blood glucose screening as compared to separate screening for the two disease entities [36].

An estimation made in United States indicated that medical screenings for diabetes mellitus, hypertension, and hypercholesterolemia in dental settings could save the health care system $13.51–$32.72 per person screened over 1 year, depending on the referral flow from dental care to health care [37]. The reported estimated socio-economic benefits in the article have been supported by others [38].

Nearly all spokespersons for the authorities or organizations replied that they had a lack of knowledge on the topic but saw opportunities for individual and societal gains with medical screening in dental settings.

This study shows that the expected impact and implications for clinicians and policy makers is to further enhance the implementation and intensify research on medical screening in dental settings.

## Conclusions

Approached authorities and organizations generally had a positive view of medical screening in dental settings but were uncertain about the concept. Further scientific knowledge and guidelines concerning the topic are needed before it can be commonly introduced and additional research on implementation strategies and long-term follow-up of medical screening are needed.
